# Spectral Decomposition and Sound Source Localization of Highly Disturbed Flow through a Severe Arterial Stenosis

**DOI:** 10.3390/bioengineering8030034

**Published:** 2021-03-04

**Authors:** Fardin Khalili, Peshala T. Gamage, Amirtahà Taebi, Mark E. Johnson, Randal B. Roberts, John Mitchel

**Affiliations:** 1 Department of Mechanical Engineering, Embry-Riddle Aeronautical University, 1 Aerospace Boulevard, Daytona Beach, FL 32114, USA; 2 Department of Biomedical and Chemical Engineering and Sciences, Florida Institute of Technology, 2930 Science Cir., Melbourne, FL 32901, USA; pthibbotuwawagam@fit.edu; 3 Department of Biomedical Engineering, University of California Davis, One Shields Avenue, Davis, CA 95616, USA; ataebi@ucdavis.edu; 4 Telecraft Engineering Inc., 1254 Mount Carmel Church Lane, Canton, GA 30114, USA; mjohnson@telecraft.com (M.E.J.); randy.roberts@telecraft.com (R.B.R.); 5 Infrasonix Inc., 1665 Lakes Parkway, Suite 102, Lawrenceville, GA 30043, USA; jmitchell@infrasonix-inc.com

**Keywords:** cardiovascular diseases, atherosclerosis, stenosis, spectral decomposition, turbulent pressure fluctuations, sound localization, noninvasive diagnostics, fluid dynamics, signal processing

## Abstract

For the early detection of atherosclerosis, it is imperative to explore the capabilities of new, effective noninvasive diagnosis techniques to significantly reduce the associated treatment costs and mortality rates. In this study, a multifaceted comprehensive approach involving advanced computational fluid dynamics combined with signal processing techniques was exploited to investigate the highly turbulent fluctuating flow through arterial stenosis. The focus was on localizing high-energy mechano-acoustic source potential to transmit to the epidermal surface. The flow analysis results showed the existence of turbulent pressure fluctuations inside the stenosis and in the post-stenotic region. After analyzing the turbulent kinetic energy and pressure fluctuations on the flow centerline and the vessel wall, the point of maximum excitation in the flow was observed around two diameters downstream of the stenosis within the fluctuating zone. It was also found that the concentration of pressure fluctuation closer to the wall was higher inside the stenosis compared to the post-stenotic region. Additionally, the visualization of the most energetic proper orthogonal decomposition (POD) mode and spectral decomposition of the flow indicated that the break frequencies ranged from 80 to 220 Hz and were correlated to the eddies generated within these regions.

## 1. Introduction

Cardiovascular diseases (CVDs) are known as the leading global cause of death, and in the United States, they account for the death of one person every 36 s [1]. This means that about 17.3 million people die annually, which is expected to grow significantly over the next decade [2]. The most common cardiovascular disease is atherosclerosis, a chronic inflammatory disease in which plaque builds up in an artery, narrowing it down and forming stenosis. It can severely reduce blood flow through the arteries. Plaques can also rupture, and consequently, a blood clot can form and block the flow, leading to the development of coronary artery disease, stroke, or peripheral artery occlusion disease, depending on the location of the atherosclerosis lesions. These diseases can cause death, paralysis, or permanent damage to vital organs [3]. In addition, based on a report published by the American Heart Association (AHA), the current CVD healthcare costs are expected to grow from $500 million per year to over $1.1 trillion by 2035, which includes the expenses related to healthcare services, medicine, and lost productivity due to disability and death [4,5]. Evaluating the disorders mentioned above, mortality rates, and associated economic impacts, detecting stenosis at an early stage is critical to reducing treatment costs before a significant amount of vascular damage occurs.

For many years, clinicians have relied on classical and, more recently, electronic stethoscopes to detect cardiovascular and pulmonary sounds associated with known diseases. Additionally, one of the most common approaches to diagnosing stenosis is angiography (arteriography), which is based on obtaining X-ray images after the introduction of a radiopaque substance into the body. Angiography is an invasive method, with the risk of bleeding and infection after the operation. The main disadvantage of both methods, and the motivation for the current study, is that they are not predictive techniques and are usually applied only after observing clinical symptoms to understand the degree of the disease. Alternative noninvasive techniques are Doppler ultrasonography and computed tomographic angiography, which can be expensive and time-consuming. Therefore, new noninvasive and low-cost diagnostic tools are essential to provide insightful details of the blood flow and, more importantly, better guidance to clinicians for the early detection and intervention of stenosis that can be applied to most patients in a more effective and optimum manner during the early stages of the disease. One of the methods used for this purpose is to record, analyze, and comprehend flow-induced mechanisms of the sounds emerging from the arterial stenosis through advanced computational modeling and signal processing techniques. It should be noted that the studies focused on the localization of the sound sources and wall pressure fluctuations, and characterizations of the resulting acoustic radiation are limited.

Abnormal conditions in the blood flow through stenosis have been investigated in many experimental and numerical studies [6,7,8,9,10]. The flow velocity increases through the stenosis, and a flow jet forms at the stenosis throat, followed by a turbulent expansion. As a result, high-frequency pressure fluctuations occur inside the vessel in the post-stenotic region. As the sound source, the flow fluctuations interact with the vessel wall, resulting in vascular sounds known as murmurs [11,12,13]. Although there are many studies on the dynamics of flows through stenosed or partially obstructed vessels, numerical studies focused on the acoustics perspective are limited. In a clinical study, with data collected from two patients with severely stenosed vessels, Lees and Dewey showed that sound waves measured at the epidermal surface may be used to diagnose stenosis [14]. In another study, the flow dynamics in a realistic stenosed vessel were investigated. They showed high-frequency vortex shedding downstream of the stenosis, with velocity fluctuations within the audible range of 100–300 Hz [15]. Mittal et al. investigated pulsatile flow in a planar channel with a one-sided semicircular constriction and found that the characteristics of the arterial murmurs are directly related to the pressure fluctuations [16]. They also studied the effect of stenosis severity on acoustic radiation using two-dimensional vessel models with 50% and 75% severities [17]. They concluded that the amplitude of acoustic pressure fluctuations increased significantly with a richer frequency content for the 75% case. A similar analysis was performed by Khalili et al. [18]. Salman and Yazicioglu numerically and experimentally investigated the vibrations of constricted arteries. They indicated that stenosis severities higher than 70% increase acoustic radiation significantly at high frequencies between 250 and 600 Hz [19,20].

It should also be noted that, during the last decade, several different acoustic-based approaches have been proposed to diagnose coronary artery diseases. However, they need to be verified by an invasive method such as angiography [21,22,23]. This issue suggests that more studies are necessary to develop strategies that can be employed to detect vascular stenosis without the need for invasive approaches. Tobin and Chang measured the wall pressure downstream of the idealized stenosis of various severities placed in a latex rubber tube and showed that the intensity of the acoustic radiation is directly related to the flow rate and the stenosis severity [24]. Accordingly, the basis of our current and future studies is to propose a noninvasive technique that can localize the anatomic origin of turbulence and correlate the frequency and amplitude of pressure to the degree, type, and progression of stenosis. In this study, a detailed analysis of blood flow through concentric stenosis at a Reynolds number of 2000 was investigated to provide complementary information regarding the structure of flow fluctuations, characteristic sounds in arterial stenosis, and the resulting acoustic pressure distribution on the vessel wall. These sounds, filtered by the vessel structure, emit through tissue and can be evaluated as a sign of stenosis on the epidermal surface. Computational studies of CVDs offer a promising modality for exploring the physics of sound generated in the body, providing valuable information that can be used as a diagnostic tool and in clinical practice.

## 2. Materials and Methods 

### 2.1. Computational Model

**Figure 1:**
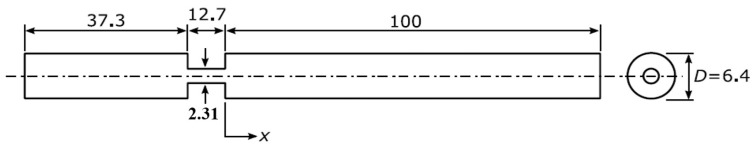
A sectional view of the flow domain. Flow is from left to right. The figure is out of scale, and the dimensions are in mm.

The exact shape of arterial stenosis is often complex and varies among patients, and it is challenging to model it accurately [25]. Hence, a simplified model of stenosis was considered in the current study, similar to previous investigations [19,26]. The schematic of the flow domain, including the idealized blunt stenosis, is shown in Figure 1. This model represents severe stenosis with a reduced flow area of 87% [9,20], calculated by Equation (1):

(1) $$Severity\;=\;\left(\frac{D^{2}-d^{2}}{D^{2}}\right)\;\times \;100\%$$

where the vessel and stenosis diameters are *D* = 6.40 mm and *d* = 2.31 mm, respectively.

The dimensions of the model are consistent with the measurements of the peripheral arteries [27,28]. Although the geometry is axisymmetric, simulations are performed as three-dimensional due to the turbulent nature of the flow. 

The arterial wall was assumed to be rigid. This assumption was made based on the results suggested in several previous studies that the wall deformation and vibration, in the presence of turbulence, are negligible in comparable experiments and more realistic computational models about the Reynolds numbers used in this study. For example, in two studies conducted by Salman et al., it was found that the wall of a stenosed artery only deformed slightly due to a significantly higher stiffness of the stenosis compared to the artery and surrounding tissue [20,29]. They concluded that similar results of the mean flow velocities and dynamic acoustic pressures were observed for the rigid wall models and by the fluid-structure interaction (FSI) analysis of the elastic walls. 

In an experimental study on the flow through a blunt stenosis, Borisyuk showed that the axial distribution of the root mean square (RMS) of the wall pressure and the location of the maximum excitation on the wall in the post-stenotic region were very close for the rigid and elastic pipes [30]. This conclusion was also valid for the lengths of flow separation, fluctuating, and reattachment regions behind the stenosis. In that study, however, it was noted that the difference in the amplitude of the RMS of the wall pressure for the elastic and rigid pipes became noticeable for Reynolds numbers higher than 9000. Mamun et al. also showed that the results of the flow velocity profiles at the peak systole through a stenosed artery with elastic or rigid walls are similar in the upstream, throat, and downstream regions [31]. This assumption was also found in many recent studies [8,16,17,32,33,34,35,36].

### 2.2. Physics and Flow Conditions

The range of velocity in the peripheral arteries is 0.066–0.642 m/s [37], corresponding to a Reynolds number range of 100–2200, depending on the vessel diameter. In this simulation, a uniform velocity of 0.3125 m/s (equivalent to Re = 2000), close to the peak velocity of the pulsatile flow in the peripheral arteries, was specified at the inlet to represent a relevant critical flow condition that shows stronger sound signals for signal processing purposes. Additionally, it was previously illustrated that the pulsatile and steady flows generate similar acoustic pressure amplitudes [38]. The entrance length upstream of the constriction is 37.3 mm, which is sufficient for the inflow to become fully developed prior to the stenosis. The flow is considered steady, because the frequency of the cardiac cycle is in the order of 1 Hz, in contrast to the frequencies of the post-stenotic fluctuations, which are usually in the range of 20–1000 Hz. The reference pressure was zero at the outlet, and the no-slip boundary condition was used at the wall boundaries. The density and kinematic viscosity of water, as a typical blood substitute used in in-vitro experiments, were set to 1000 kg/m^3^ and, 10^−6^ Pa·s, respectively.

A numerical analysis was conducted using Simcenter STAR-CCM+ (2020.1.1, CD-Adapco, Siemens PLM, Plano, TX, USA) with a dynamic Smagorinsky turbulence model to simulate the transitional flow with appropriate scale resolving simulation (SRS) modeling. This model was suggested in the literature for the simulation of flows inside stenotic vessels [8,39,40]. A time step of 2.5 × 10^−5^ s was evaluated to be sufficient, through a time independence study, to ensure the accuracy of the turbulent transients and a Courant number close to 1. In addition, the time step convergence was less than 10^−4^, which is particularly important with SRS models [41]. The initial velocity was set to a value close to the inlet velocity to reduce the initial residual errors. The governing equations were discretized using second-order central discretization in space and second-order implicit discretization in time.

### 2.3. Proper Orthogonal Decomposition (POD) Analysis

In the current study, proper orthogonal decomposition (POD) was used to investigate the coherent flow structures in the flow domain. POD decomposes the time-varying flow field into spatial and temporal parts. While the spatial part describes the modes that represent the coherent flow structures in the flow, the temporal part delivers the time evolution of these modes. Hence, POD can provide useful insight into the acoustic sources in the flow. Certain studies have employed POD to study the flow structures and sound sources in similar flow geometries to the current study, which involve constrictions [42,43,44]. POD can be explained using Equation (2):

(2) $$p\left(X,t\right)≅\overset{M-1}{\underset{i=0}{\sum }}\mu_{i}\left(t\right)\varphi_{i}\left(X\right)$$

where a time-varying quantity, p(X,t), is represented as a summation of linearly independent (i.e., orthogonal) mode shapes $\varphi_{i}(X$) multiplied by their time-varying amplitudes $\mu_{i}\left(t\right)$. 

According to the energy content of POD modes, these modes are ordered, which means that the first modes are the most energetic ones. When M→∞, the summation of the mode shape perfectly represents p(X,t). In Equation (2), X denotes the spatial coordinates (x, y, z). Here, we employed a singular value decomposition (SVD) method to compute POD, and these calculations were done in MATLAB (2020b. The MathWorks, Inc., Natick, MA, USA). A detailed description of this POD calculation procedure can be found in a previous study [44]. In the current work, POD was performed for the pressure values in the flow domain solved in the last second of simulation and was recorded with the frequency of every 0.0008 s, corresponding to a Nyquist frequency of 625 Hz. The frequency of 625 Hz was selected, as it was observed that, for stenosis, severities higher than 70% acoustic radiation at high frequencies of 100–600 Hz have the highest energy. In addition, the analysis of higher frequencies is mostly related to very small-scale eddies outside of the fluctuating region with low energies, which could also lead to high computational costs due to our semi-coupled computational fluid dynamics (CFD) signal processing technique.

### 2.4. Mesh

The use of scale resolving simulation (SRS) turbulence models for wall-bounded flows requires a high-quality mesh, and when creating such mesh, it is essential to maintain y^+^ ≤ 1 [10,45]. Mesh should be finer in those areas where high physical gradients are present. Therefore, to increase the accuracy of the flow solution and capture the high flow fluctuations, a solution-based mesh refinement method was used based on a flow parameter. In this study, turbulent kinetic energy (TKE) was selected, since it is an indication of flow fluctuations and energy of sound sources. This mesh refinement was accomplished through the following steps: (1) generate an initial coarse mesh on the geometry, (2) solve a steady-state flow and use TKE to threshold and identify the cells that require refining, (3) create a field function to set a new cell size for the flagged cells for refinement, (4) create a refinement table for the entire domain with the refinement field function as the scalar and extract the values, and (5) add the refinement table to your volume mesher and regenerate the volume mesh. This way, the mesh was optimally refined based on an important flow parameter in the region of interest to avoid unnecessary mesh cells throughout the domain and to reduce the computational costs. This semiautomated refinement method reduced the computational times by about 30% compared to a manual meshing method based on the different predefined regions (inlet, stenosis, fluctuating zone, reattachment, and laminar flow regions).

The minimum mesh cell size, in step 3 of this mesh refinement method, was determined through a grid independence study for four different mesh configurations with total number of mesh cells of approximately 400 k, 700 k, 1.4 M, and 2 M. This was conducted to find an optimum mesh density and resolution while keeping the Courant number close to 1 and y^+^ ≤ 1. After an evaluation of the mean velocity in flow direction along the pipe, the mesh with the maximum cell size of 0.144 mm and total number of mesh cells about 1.4 M was selected. This was also determined suitable for the large eddy simulation (LES) simulations after calculating the ratio of the cell size to the minimum Kolmogorov length scale obtained in the fluctuating region (1D to 4D downstream of stenosis). A ratio of below 20 was attained, which is within the maximum allowable range suggested by [46].

It should be also noted that an accurate prediction of the pressure drop in flows with separation depends on resolving the velocity gradients normal to the wall. Prism layers allow the solver to resolve the near-wall flow more accurately. In this study, 10 prism layers with a total thickness of 0.059 mm and layer stretching factor of 1.35 were generated near the boundaries to resolve the velocity gradients normal to the wall.

### 2.5. Validation

**Figure 2:**
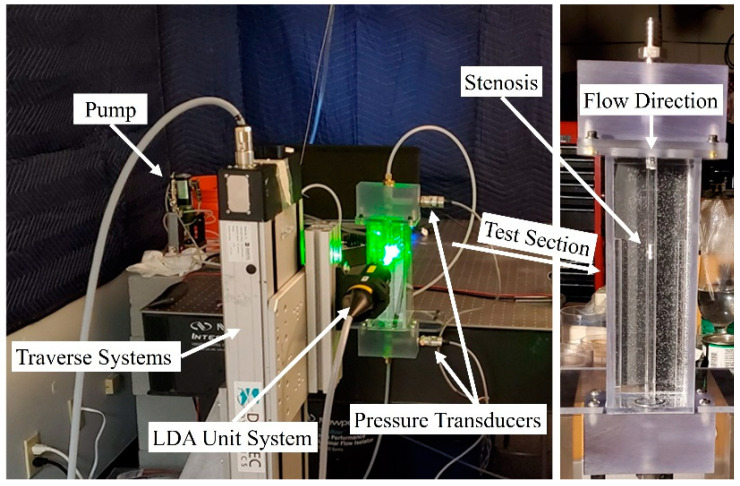
Experimental setup of the Laser Doppler Anemometry (LDA) axial velocity measurements for a constricted pipe representing arterial stenosis.

In the current study, Laser Doppler Anemometry (LDA), as a nonintrusive method that does not interfere with the flow field and sound generation, was used to measure the velocity at different locations in the upstream and post-stenotic regions to validate numerical results. Figure 2 displays the experimental setup for a constricted pipe (a simplified model of a stenosed artery). The velocity measurements using an LDA system (Dantec Dynamics A/S., Skovulunde, Denmark) were performed for 30 s at each location with sampling frequencies of more than 1000 Hz.

**Figure 3:**
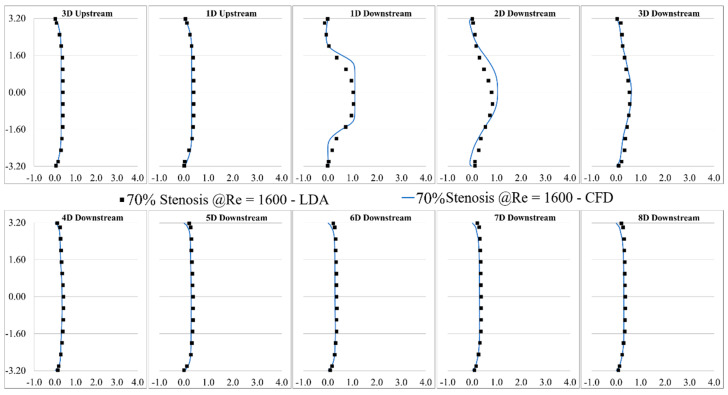
Validation of the computational results of 70% stenosis at Re = 1600 compared with the LDA measurements.

**Figure 4:**
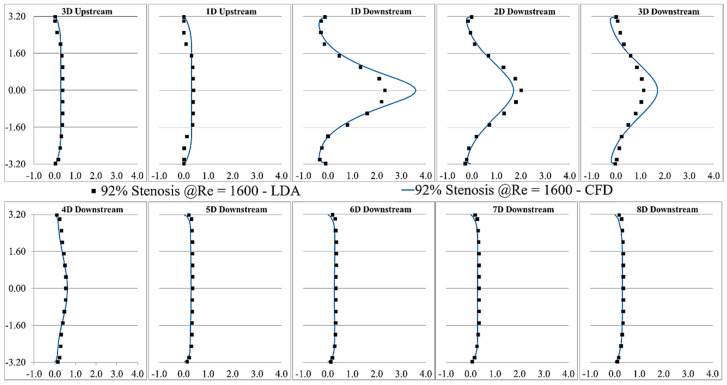
Validation of the CFD results of 92% stenosis at Re = 1600 compared with the LDA measurements.

LDA measurements were performed at eight different locations downstream of the constricted region, separated each by one diameter. The validation was done for several severity levels and Reynolds numbers, such as 70% and 92% stenosis at Re = 1600, as shown in Figure 3 and Figure 4. Note that correlation of the sound sources with the progression of the stenosis severity at different Reynolds numbers and for different types of stenosis is the objective of another study that we will conduct in future works after the current analysis.

**Figure 5:**
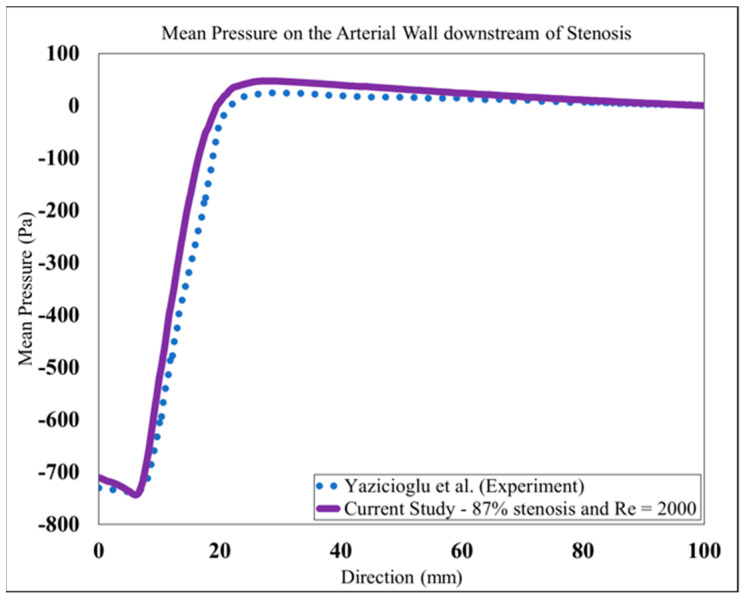
Mean pressure on the arterial wall in the post-stenotic region compared to [26].

Based on the study of mesh independency, the mean wall pressure variation along the wall in the post-stenotic region was also examined. The final result of the mean pressure at the wall was shown in Figure 5. It was compared with a previous experimental study [26] in which a catheter-type pressure transducer was used, which was able to collect the mean and fluctuating pressure signals within the vessel lumen. The results showed good agreement for both the wall pressure differences and the location of the maximum pressure. As the blood flow entered the expansion region, the pressure distribution changed significantly, leading to a wall pressure difference of about 700 Pa between the stenosis and flow domain exits. Additionally, the mean wall pressure reached its maximum value at about 25 mm (or, x = 4D); after which, it decreased gradually to the exit.

The validation results showed that the LES turbulence model had a good agreement with experimental results, especially in the regions where the flow separation occurred, and the flow experienced the highest fluctuation about 1D to 4D downstream of the stenosis.

## 3. Results and Discussions

**Figure 6:**
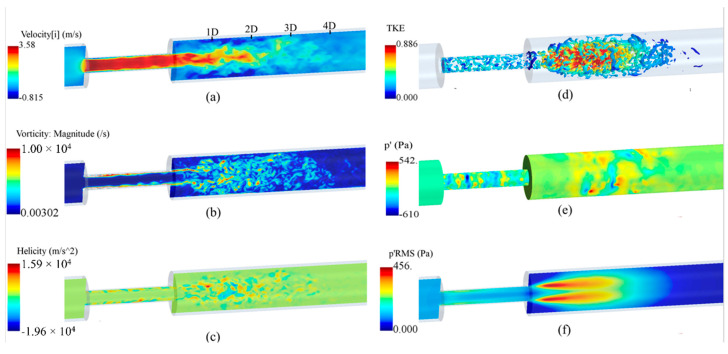
Instantaneous flow parameters: (**a**) axial velocity; (**b**) vorticity; (**c**) helicity; (**d**) the turbulent kinetic energy (TKE) on the isosurfaces of Q-criterion; (**e**) wall pressure fluctuations, and (**f**) root mean square (RMS) of the pressure fluctuations at the middle cross-section of the flow domain.

To get an overview of the solutions, some instantaneous flow parameters at the middle cross-section of the flow domain are shown in Figure 6. The flow solutions were obtained after the mean flow reached a steady state, i.e., after about 2.4 s of solution (or five times 0.48 s, which was the time required for a fluid element to travel from the inlet to outlet). Additionally, the mean velocity components and pressure were obtained by averaging over the flow field for the last 3.4 s of simulation to deliver more accurate results than the shorter time-averaging presented in some previous studies. It was considered that the transient effects on the mean flow parameters would diminish before recording all flow parameters, such as the mean and fluctuating flow parameters.

As shown in Figure 6a, the axial velocity significantly increased inside the stenosis due to a reduction in flow area, forming a flow jet at the exit, and, in recirculation regions, that led to a severe pressure drop. The maximum velocity in the flow direction elevated to nearly 3.60 m/s, which was about 12 times larger than the inflow velocity. At 1D downstream of the stenosis, the shear layers around the flow jet became unstable and rolled into vortices that interacted with the wall and with each other, breaking into smaller eddies. This is the main source of wall pressure fluctuations. The region between 1D to 4D downstream of the stenosis contained the highest fluctuations in the post-stenotic region. The length of the recirculation region was about x = 3D, consistent with previous studies [30,47]. More detailed discussions on this “fluctuating zone” that consists of the sound source potential to transmit through the arterial wall and tissue are included in the next sections. Around x = 4D, vortical structures start to lose their strength, and whereafter, fully developed turbulent flow were observed. In Figure 6b, the vorticity at a cross-section of the model is displayed and limited to 10^4^ s^−1^ to clearly present the flow fluctuations in the post-stenotic region. The LES model could accurately predict the fluctuations and capture smaller eddies in this region. Helicity is proportional to the flow velocity and vorticity, and it indicates the potential for the development of a helical flow. Figure 6c suggests that intense vortical structures started to appear inside the stenosis where the flow was mostly unstable close to the stenosis wall (plaque surface). 

A similar flow pattern was observed in the post-stenotic region in which high-energy vortices were shown. To distinctly illustrate the vortical structures with the highest energy, the turbulent kinetic energy (TKE), representing the energy of flow fluctuations, was mapped on instantaneous vortex cores visualized using the isosurfaces of Q-criterion, Figure 6d. It highlighted that flow fluctuations started within the stenosis close to the wall and spread around in the expansion region from 1D to 4D downstream of the stenosis before the flow reattachment and stabilization occurred. In contrast to the region inside the stenosis, flow fluctuations are more concentrated close to the middle of the domain in the post-stenotic region. Compared with the results in [8,18,48,49], depending on many variables, such severity and length of the stenosis, these observed phenomena can alter, which requires further investigation in future studies. Figure 6e,f show the pressure fluctuations on the wall and the root mean square (RMS) of the pressure fluctuations on the middle cross-section, respectively, expressing the potential of transmission of fluctuations through the vessel wall. Again, high-energy pressure fluctuations existed in the stenosis closer to the wall, with the presence of the turbulence due to the length of the stenosis. These high-energy fluctuations in the stenosis usually lead to higher wall shear stresses and extreme interactions of flow with the plaque surface that can cause a rupture, thromboembolism, and stroke occurrence. There is almost no study in the literature that focused on the fluctuations and the generation of sound sources inside the stenosis.

**Figure 7:**
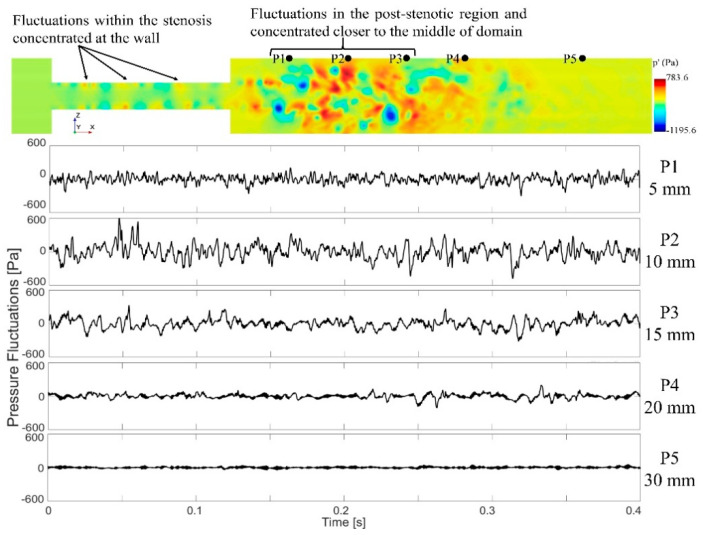
A cross-sectional view of the pressure fluctuations and time series data of the wall pressure fluctuations on the sample points at different locations downstream of the stenosis. Note that only a part of the flow domain close to the stenosis is shown in this figure.

Cardiovascular sounds captured with a stethoscope are ambient pressure fluctuations known as acoustic pressure fluctuations, which are correlated with the vibration of the epidermal surface as a result of propagated sound waves from the vessel through tissue. Pressure fluctuations, especially on the internal arterial wall, are the focus of this study. They account for the primary sources of sound due to the structural response of the surrounding tissue. Figure 7 shows the instantaneous pressure fluctuations in the middle cross-section. Pressure fluctuations are first generated inside the stenosis as small vortices are initiated at the wall and then are propagated towards the post-stenotic region. Due to the flow area expansion and jet separation in the post-stenotic region, the flow becomes more unstable, leading to higher-energy turbulent fluctuations within the fluctuating zone, also found in [8]. The time history of fluctuating pressure was recorded at 41 nodes on the vessel wall in the post-stenotic region, each separated 2.5-mm apart, for the last 3.4 s of the simulations at every time step. The energy of the pressure fluctuations was the highest through the stenosis, close to the wall, as well as 1D to 4D downstream of the stenosis. It was observed that the energy of the turbulent pressure fluctuations started to dissipate after 4D downstream of the stenosis, where eddies further break into smaller ones.

**Figure 8:**
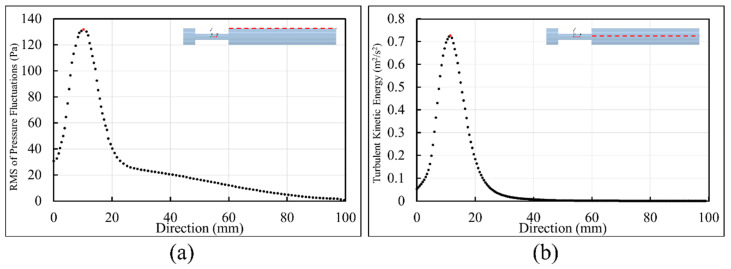
Point of maximum excitation in the post-stenotic region by analyzing (**a**) the RMS of the pressure fluctuations on the wall and (**b**) TKE on the stenosis centerline.

To localize sound sources, it is essential to find the point of maximum excitation in the post-stenotic region. Variation of the RMS of the pressure fluctuations along the wall and TKE along the centerline of the stenosis are shown in Figure 8. With the existence of flow recirculation, the RMS of pressure fluctuations started around 30 Pa at the exit of stenosis on the wall and increased to a maximum value of about 130 Pa at 11.5 mm downstream of the stenosis (or about x = 2D), as shown with the red dot, Figure 8a. This is within the region of interest with the maximum arterial wall fluctuations. Similar results were concluded in a previous experimental study [24]. After the peak value, as the energy of fluctuations dissipated, the RMS of the pressure fluctuations was down to around 20 Pa at about x = 40 mm, and it gradually decreased to zero along the wall.

Similarly, in Figure 8b, TKE increased from the exit of the stenosis on the centerline and reached the highest level at about the same point shown with the red dot. TKE reduced rapidly as the flow became reattached and laminarized. From x = 30 mm to the exit, the turbulent flow became stable and fully developed further downstream of the stenosis, as shown in Figure 3 and Figure 4.

**Figure 9:**
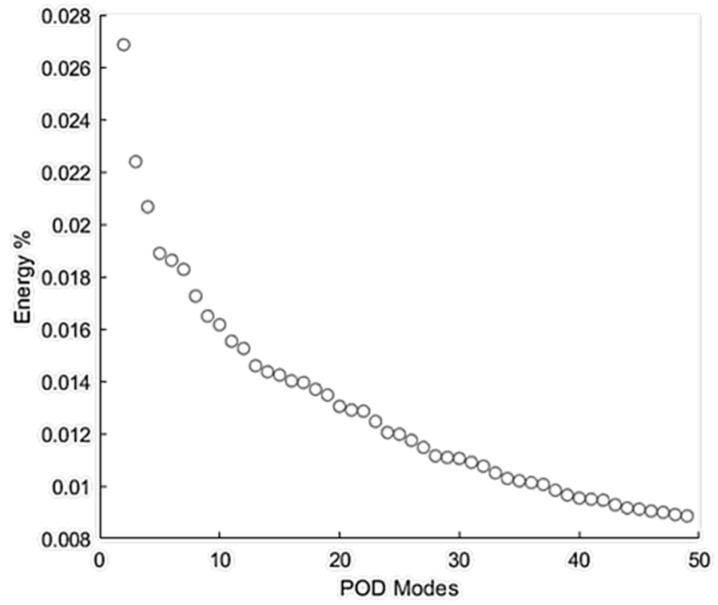
Energy % distribution of the first 50 proper orthogonal decomposition (POD) modes of flow through the stenosis.

Figure 9 shows the energy distribution of the first 50 POD modes. POD Mode 0 is related to the mean flow with the highest energy, which was excluded from this figure. These results showed that POD mode 1 (i.e., the next most energetic mode) contained about 2.7% of the total energy of all the modes. Although an analysis of higher-order (i.e., low-energy) POD modes may unveil information on important flow fluctuations with specific higher frequencies related to the turbulence or geometry characteristics [43], the scope of this paper limits the focus to analyze coherent structures with the highest energy fluctuations (i.e., POD mode 1) that may help identify the region(s) with high-energy acoustic sources.

**Figure 10:**
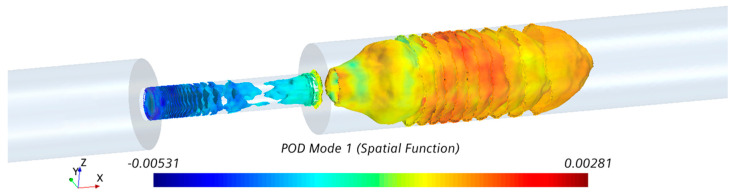
Visualization of POD mode 1 with the highest energy in the stenosis and post-stenotic region.

A visualization of POD mode 1 (i.e., $\varphi_{1}\left(X\right)$) in the fluid domain is shown in Figure 10. Ring-like flow structures originating from the upstream edge of the constriction were observed. These ring-like structures were distorted in the middle of the stenosis, which can be correlated to the length of the stenosis. Additionally, organized (bellow-shaped) flow structures were observed in the post-stenotic region within the fluctuating zone, which diminished when the flow reached further downstream and became stable. The POD analysis complemented the pressure fluctuations results (Figure 6e,f and Figure 7), which showed a part of the high-pressure fluctuations concentrated at the stenosis wall. The spectra of the POD mode (calculated using respective time evolution $\mu_{1}\left(t\right)$) showed a dense spectrum without any clear peaks. These results suggested that high-energy sound sources (which may have the potential to propagate to the epidermal surface) are likely to be generated both inside the stenosis and post-stenotic region, highlighting the importance of the study of the flow fluctuations inside stenosis, as most studies have only focused on the flow in the downstream section [19,20,26].

**Figure 11:**
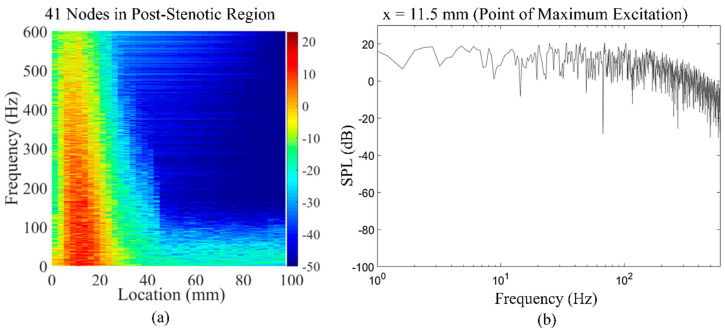
(**a**) Acoustic spatial-frequency map of the post-stenotic region and (**b**) fast Fourier-transform (FFT) of the wall pressure fluctuations at the point of maximum excitation (x = 11.5 mm) in the post-stenotic region.

The extracted time series of the wall pressure (Figure 7) on 41 nodes along the post-stenotic vessel wall were recorded at a sampling frequency of 8 kHz. The data was then postprocessed using MATLAB to transform into the spectral domain by performing Hanning window filtering and fast Fourier-transform (FFT) and providing an acoustic spatial-frequency map of the post-stenotic region shown in Figure 11a. The acoustic pressure amplitudes (or sound pressure levels (SPLs)) were converted to a logarithmic scale using Equation (3):

(3) $$p\left(dB\right)=20\log_{10}\left(\frac{P}{p_{ref}}\right)$$

where P is the computed pressure in the simulation in pascals, p(dB) is the sound pressure level converted into decibels, and $p_{ref}$ is the reference pressure set to 1 Pa, similar to previous studies [24,26]. Cardiovascular systems generate acoustic waves in a frequency range of 20–1000 Hz [50,51]. It can also be observed that peak frequencies (up to 600 Hz) with the highest energy exist 1D–4D downstream of the stenosis, while the energy content of the sound sources drop significantly with the increasing frequency and distance from the stenosis. Figure 11a shows the frequency content of the acoustic pressures at the point of maximum excitation in the post-stenotic region. At this point, which is around x = 11.5 mm, the energy of the wall pressure fluctuations was higher than the other points on the vessel wall. 

The energy of the disturbed flow decreases with the increase in the distance from the obstruction. The variation in the frequency spectral of the flow can characterize the energy of eddies of different sizes in the post-stenotic region, which can help clinicians determine the location of the stenosis relative to the point of measurements.

**Figure 12:**
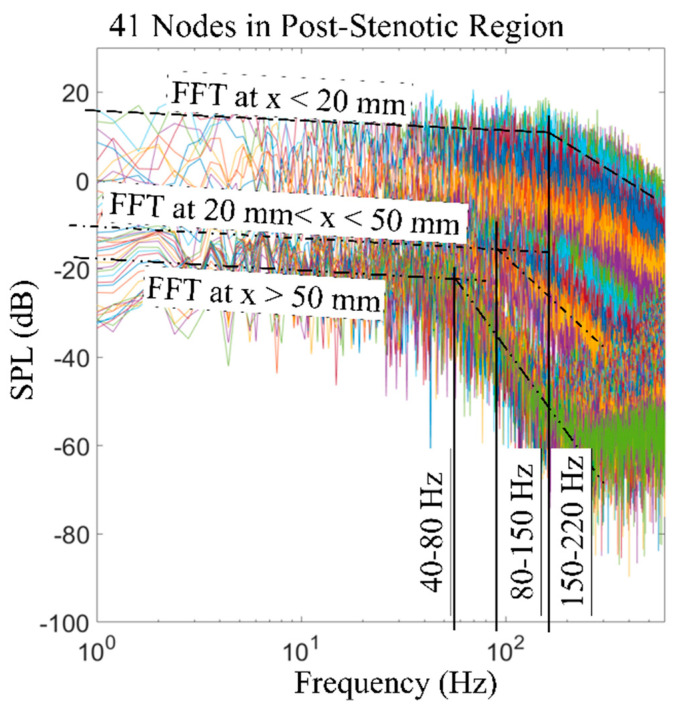
FFT of the wall pressure fluctuations at 41 nodes along the wall in the post-stenotic region. Break frequencies and ranges of locations are included. SPL: sound pressure level.

Figure 12 shows an overview of the sound pressure level variations with the frequency of the acoustic pressure fluctuations on all 41 nodes at the post-stenotic wall. The purpose of this plot is to highlight that frequency and energy contents of turbulent wall pressure fluctuations strongly depend on the flow structure in different regions of the domain. It was observed that the SPL was the maximum in the fluctuating and recirculation zones, followed by the flow reattachment region. Then, the energy of the wall pressure fluctuations dropped significantly after the flow became stable. The dashed black lines drawn at different angles on this plot display the general initial slope of the frequency spectrums. The slope of the spectrum was similar for some of the points at the wall. Therefore, based on the results, the frequency spectrums were put into categories based on the location of their corresponding point at the wall: x < 20 mm, 20 mm < x < 50 mm, and x > 50 mm. In addition, the slopes changed at a specific frequency. This is known as the break frequency, indicating the frequency at which the energy of the pressure fluctuations in the flow turns into the acoustic energy [16,50,52]. The break frequency is due to an energy transfer from a turbulent flow to acoustic radiation when the large eddies break into smaller ones with high-energy fluctuations. As the sound waves transmit from a highly disturbed blood flow in unhealthy vessels through the wall and surrounding tissue, understanding of the sound characteristics, such as the break frequency, is essential. Sound waves with higher energy generated at break frequencies are more potentially detected at the epidermal surface by utilizing noninvasive techniques. Additionally, the high-frequency fluctuations can propagate through the thorax in a form of pressure waves that have a speed in the order of 1000 m/s [53]. The turbulence-induced sounds recorded on the chest wall surface are widely referred to as heart murmurs, which can be detected with an analog stethoscope. With the use of advance sensors and signal processing, these high frequency sounds can be scrutinized to extract sound features to diagnose coronary stenosis. The 2D sensor array on the chest surface can be used to passively localize the sound source, as shown in previous studies [54,55].

**Figure 13:**
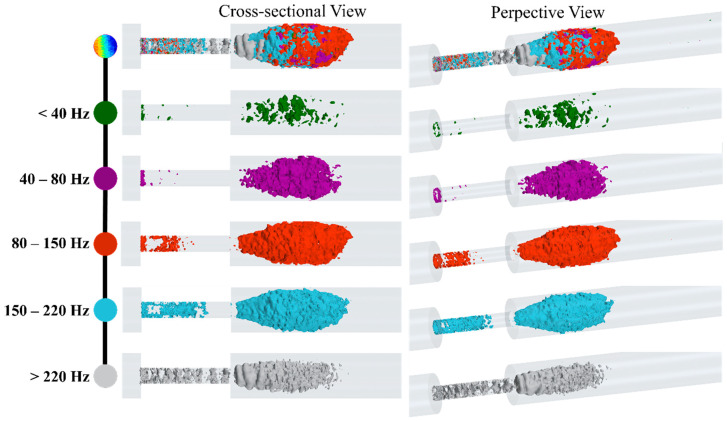
Snapshots of high-energy fluctuations are shown with isosurfaces of the RMS of acoustic pressure for different bandwidths.

To visualize the spectral decomposed acoustic pressures, we applied temporal filtering to the pressure fluctuation, $p^{\prime }\left(X,t\right)$, for the last 0.2 s of data. Here, the time series of $p^{\prime }\left(X,t\right)$ values at each mesh node was forward-backward-filtered through a bandpass sixth-order Butterworth filter with specified cutoff frequencies. These filters were designed using the MATLAB signal processing toolbox (2020b. The MathWorks, Inc., Natick, MA, USA). Acoustic pressures were visualized using isosurfaces of the RMS of filtered pressure fluctuation, $p_{rms}^{\prime }\left(X,t\right)$, for different frequency ranges, as shown in Figure 13. Such a visualization can help the acoustic source localization of specific frequencies in the flow domain. These frequency ranges were selected such that the focus was given to the break frequencies of the spectra measured in the post-stenotic region at different points on the wall, as shown in Figure 12. These results indicated that all frequencies are mainly generated inside the stenosis and the post-stenotic region, up to x = 4D. Aligning with our flow analysis results shown in Figure 6 and Figure 7, which indicated the existence of turbulent fluctuations inside the stenosis and post stenotic regions, overall, it can be concluded that the turbulence generates broadband acoustic sources in these regions. 

At lower frequencies (<40 Hz), the fluctuations related to larger eddies are mostly around the flow jet and separation regions. As the shear layers became unstable and large eddies cascaded into smaller eddies, the isosurfaces of $p^{\prime }\left(X,t\right)$ were expanded. For higher frequency ranges, especially between 80 Hz to 150 Hz, the fluctuations consist of smaller eddies that were extended up to 4D downstream of the stenosis. Interestingly, for higher frequencies (i.e., >220 Hz), fluctuations in the stenosis had more contribution to the frequency spectrum, and more organized ring-like isosurfaces were observed inside the stenosis. Adversely, these increased fluctuations at the plaque surface can attenuate or amplify depending on the stiffer plaque material properties (which may include calcifications) and the mechanical coupling of the artery to the body (which can induce vibrational modes). Further, such intense variations in the pressure and stress at the stenosed region can cause a fracture in atheromatous plaques, leading to embolism. Hence, this previously unexplored phenomenon is worthy of investigation, as it may be of potential diagnostic value.

Details of the flow pressure fluctuations and the associated sound sources in the post-stenotic region are essential in phonocardiography. The results shown here provided helpful information on the spectral structure and spatial characteristics of the pressure fluctuations. These pressure fluctuations contribute to the acoustic generation as a result of the structural response of the surrounding tissue.

## 4. Conclusions

Comprehending flow-induced mechanisms of the sounds propagated from arterial stenosis can lead us to predictive techniques for diagnosing atherosclerosis before it progresses to severe cases. In this study, a detailed investigation was performed to localize the high-frequency turbulent pressure fluctuations in the stenosis and post-stenotic regions, with more focus on the vessel wall. The results of this study are listed as follows:

1. The analysis of the flow solution showed that the flow velocity increased significantly inside the stenosis and became unstable, leading to significant pressure fluctuations at the plaque surface. It indicates the possibility of increased fluid-solid interactions and subsequent excitation of the vessel wall;
2. As the flow jet entered into the expansion region, flow separation occurred at x = 1D, where large eddies started to cascade into smaller eddies with higher rotational energies, up to 4D downstream of the stenosis. These eddies were the origin of flow-induced acoustic radiation, which was mostly concentrated around x = 11.5 mm, as the point of maximum excitation. It is important to avoid this region and move the measurement probe further downstream (i.e., x > 4D) of the vessel during coronary catheterization measurements such as the fractional flow reserve (FFR) for accurate readings;
3. The analysis of the spectra of the recorded pressures at the wall also showed that the most energetic POD mode of the flow appeared in the same regions, which complimented the results from the fluid dynamics analysis;
4. The spectral decomposition of the pressure fluctuations showed broadband acoustic sources distributed in the same region (1D to 4D) generated from turbulence.
5. Low-frequency (i.e., <40 Hz) acoustic fluctuations were observed mostly around the flow jet and the separation regions, which were correlated with larger eddies. The break frequency, as a characteristic of the sound transmitted through the vessel wall and surrounding tissue, was considered in the temporal filtering of the acoustic pressure;
6. At higher frequency ranges between 80 Hz to 220 Hz, the fluctuations related to smaller eddies appeared at the entrance of the stenosis and, in the middle of the fluctuating region, extended up to 4D downstream of the stenosis;
7. The results also showed organized ring-like isosurfaces of fluctuations inside the stenosis at high frequencies over 220 Hz.

These results are significantly important when exploring the sound characteristics of stenosed vessels to develop new noninvasive diagnostic methods for early detection of the disease. Further, similar studies on several stenosis will also expand the current knowledge to determine the degree of vessel obstruction without the use of any invasive diagnosis techniques such as arteriography.

## 5. Application Feasibility

Merely the existence of acoustic frequencies generated due to turbulence in the stenosed coronary arteries allows its use as a diagnostic tool. These frequencies (ranging from ~100 to 1000 Hz) differ from other frequencies observed over the chest surface associated with respiration, cardiac blood flow, and cardiac vibrations (which are less than ~50 Hz) [26,56]. Hence, the detection of the propagated acoustics from stenosed coronary arteries on the chest surface can be used as a noninvasive diagnostic tool to identify the disease. Furthermore, the in-depth analysis of these sound features and their spatial distribution can contain valuable information about the stenosis geometric details and its location, which are of diagnostic value. To serve this purpose, we focused on analyzing the genesis of high-energy turbulence-induced acoustics (which have the potential to propagate to the chest surface) and their spatial distribution on the vessel wall. Moreover, we used spectral decomposition techniques to identify flow structures related to specific frequency ranges of the measured sound spectra, which provided an enhanced interpretation to passively localize the stenosis. As the coronary arterial tree is not located right beneath the epidermal surface, the feasibility of the method heavily depends on the sound transmission through the thorax. Although it is evident that turbulence-generated coronary sounds can be heard over the chest surface (widely referred to as heart murmurs), the acoustic transmission can attenuate based on the parameters related to the thickness and stiffness of the chest muscle, fat layer, and arterial wall. 

In a practical scenario, the accuracy of the method is also determined by the sound acquisition procedure. It is ideal to develop the sensor technology (e.g., contact microphone) with high sensitivity in the target frequency range (i.e., ~100–1000 Hz) with a good signal-to-noise ratio. It can be achieved with an improved sensor design and advanced signal processing techniques. The use of a sensor array on the chest surface that can measure the spatial distribution of the propagated acoustics can be used to localize the stenosis using source localization algorithms such as beamforming [54]. Besides, the sound can be measured via more advanced methods, such as the Doppler mode of conventional ultrasound imaging, to directly capture the high-frequency vessel wall vibrations on the heart wall surface [57]. The accurate implementation of a sound acquisition strategy, along with an enhanced understanding of the genesis of turbulent flow-induced sounds, as described in this study, can help to implement artificial intelligence-based diagnostic tools. This proposed methodology can assist clinicians to localize coronary stenosis by extracting features from measured acoustic spectra on the chest wall surface.

We should highlight that this study clearly demonstrated the existence of a highly turbulent flow inside the stenosis and in the post-stenotic region using several state-of-the-art CFD signal processing methods such as POD analysis and the spectral and flow decompositions techniques. Turbulence significantly affects the pressure drop and wall shear stresses, leading to adverse conditions in the cardiovascular system—more specifically, on the endothelial tissue of artery walls. The turbulent-induced increase in shear stresses can result in arterial murmur sounds and post-stenotic dilatation. In addition, shear stresses and pressure fluctuations close to the wall can cause the fracture of plaques, which may further lead to embolization and stroke. In some previous studies, the role of turbulent flow, with pressure fluctuations and high shear stresses on the inner artery wall, on the increase in the risk of blood damage, platelet activation, and clotting was also explained extensively [37,58,59,60,61,62,63].

## 6. Future Works

Finding the correlations between the sound sources and variables such as the stenosis shape, severity, and length, as well as the flow conditions, requires further investigation. The current study is motivated by previous works that have shown the existence of these sounds over the chest surface from in vivo and in vitro studies [11,14]. In the current study, we provided complementary information on the structure of the flow fluctuations, acoustic pressure distribution on the vessel wall, and characterizing the sound sources in the stenosis using a state-of-the-art CFD signal processing technique. In future studies, additional variables will be investigated to find the characteristic sounds of different cases to suggest a general noninvasive and predictive method for the early detection of stenosis. Some of the future studies will include:

- The modeling of flow-induced acoustics in patient-specific models derived from medical imaging. Although the simplified concentric stenosis geometry can help derive qualitative conclusions, the patient-specific irregular stenosis profiles can lead to specific alterations in the generated sounds;
- An acoustic analysis of the progression of stenosis at different levels of severity. The understanding of sound signatures of a stenosis at different stages of the disease can assist to develop an algorithm for the early detection of the stenosis;
- A pulsatile patient-specific flow. The steady flow assumption in this study represented the peak systole of the pulsatile flow. However, the pulsatile flow, with the turbulent diffusion during the diastole with lower flow rates, generates more homogeneous spectra;
- Modeling of elastic wall structural response. Although we verified the use of a rigid wall for this study according to the literature, we should agree that, for the studies focused more on the correlation of hemodynamic parameters with the gradual development of stenosis size and the interactions between the flow and the artery wall, especially with different stiffness of the stenosis, artery, and surrounding tissue, the modeling assumption of an elastic wall becomes more relevant and acceptable.

It should be noted that, with the use of state-of-the-art sensor technology, advanced computational modeling, and signal processing techniques, it is possible to isolate the flow-induced sounds generated in a constricted vessel from other sources of sounds from within the body. In addition, we are planning to expand the current methodology and technique to a CFD-FEA signal processing approach to analyze the progression of a stenosis. In the CFD-FEA signal processing approach, we will consider the artery, artery wall, and the surrounding tissue with the associated material properties and focus on the sound generation due to the disturbed, highly turbulent flow and the propagation of the sound waves through the artery and surrounding tissue to record the acoustic pressure at the epidermal surface.

## Data Availability

The data presented in this study are available on request from the corresponding authors. The data are not publicly available since we are in the technology development and funding proposal preparation phases of our research project.
